# A Positive Versus Negative Interaction Memory Affects Parole Officers’ Implicit Associations Between the Self-Concept and the Group Parolees

**DOI:** 10.3389/fpsyg.2022.787583

**Published:** 2022-07-28

**Authors:** Marina K. Saad, Luis M. Rivera, Bonita M. Veysey

**Affiliations:** ^1^Department of Criminology and Criminal Justice, Fairleigh Dickinson University, Hackensack, NJ, United States; ^2^School of Criminal Justice, Rutgers University-Newark, Newark, NJ, United States; ^3^Department of Psychology, Rutgers University-Newark, Newark, NJ, United States

**Keywords:** empathy, parole, self-expansion, implicit social cognition, criminal identity

## Abstract

**Background:**

Parole officers are one of many actors in the legal system charged with interpreting and enforcing the law. Officers not only assure that parolees under their supervision comply with the terms of their release, but also monitor and control parolees’ criminal behavior. They conduct their jobs through their understanding of their official mandate and make considered and deliberate choices while executing that mandate. However, their experiences as legal actors may impact their implicit cognitions about parolees. This experiment is the first of its kind to examine implicit (i.e., automatic) associations between the self and parolees among actors of the legal system.

**Objective:**

The present study examines the implicit cognitive consequences of the quality of the parole officer-parolee relationship from the perspective of the parole officer; specifically, whether parole officers who are reminded of positive experiences with parolees implicitly associate more with the group parolees than those reminded of a negative experience. In addition, we explore the moderating effects of parole officers’ subjective professional orientation and identification.

**Method:**

Eighty-four New Jersey parole officers participated in the study. First, an experimental manipulation of either a past positive or negative experience was administered *via* a writing task. Participants then completed an Implicit Association Test (IAT) to measure associations between the self-concept of parole officers with parolees who are part of the group criminal, followed by measures of professional orientation and identification.

**Results:**

Participants who were reminded of a positive experience with a parolee exhibited stronger associations between self and the group parolee when compared to those who were reminded of a negative experience. Neither professional orientation nor parole officer group identification were related to implicit associations and did not moderate the effect of the past experience reminder on implicit associations.

**Conclusion and Implications:**

Implicit cognitions of parole officers may influence their behaviors and interactions with those whom they supervise. Positive reminders affect implicit self-associations with parolees presumably *via* empathy, which is known to affect the quality of therapeutic and supervision relationships; thus, theoretically, leading to improved outcomes for both officers and parolees.

## Introduction

We say, “justice is blind,” yet we know that extra-legal factors (e.g., race, gender) are often associated with various law-related decisions, including arrests (e.g., Fielding-Miller et al., 2020), verdicts and sentencing (e.g., Cohen and Yang, 2019), and parole release decisions (e.g., Huebner and Bynum, 2008). Much of the extant research assumes that decisions are arrived at in a thoughtful considered way, and, despite this, that bias often influences these decisions. Bias, of course, can be a conscious cognitive process, and, importantly, it also can operate automatically outside of conscious awareness (for reviews, see Gawronski and Payne, 2011). Automatic or implicit cognitions, such as bias, also may affect interactions between actors in the justice system (e.g., between citizens and police officers, parolees and parole officers). Only recently has implicit social cognition been studied in samples of justice-involved people (Rivera and Veysey, 2015, 2018; Veysey and Rivera, 2017). The present study is the first to our knowledge to examine implicit self-cognitions among actors in the justice system. Using a sample of parole officers, we investigate the implicit association of the self with the group parolee following a positive (versus negative) interaction with a parolee. We suggest that this effect occurs *via* self-expansion, a mechanism that supports positive downstream outcomes for both parole officers and parolees.

### Parole in the United States

At year-end 2019, there were 878,900 individuals under parole supervision (Oudekerk and Kaeble, 2021). Historically, and to a large degree today, the role of the parole officer is to assist in the successful reentry of individuals who have been released from prison while monitoring their behavior and the terms of their release (e.g., maintaining employment and stable housing, abstaining from alcohol and drugs); in sum, responding to their criminogenic and non-criminogenic needs to ensure community safety (Seiter, 2002; National Research Council, 2008).

In the parole officer-parolee relationship, parole officers may function as agents of change who encourage parolees to comply with the conditions of parole, engage in specialized programs, and promote pro-social behaviors and identities (Gibbons and Rosecrance, 2005; Abadinsky, 2009) or alternatively, parole officers may foster an adversarial relationships with their parolees (Ireland and Berg, 2007; Morash et al., 2014; Chamberlain et al., 2017). The effectiveness of the parole officer-parolee relationship is dependent on the parole officer’s ability to create and maintain positive relationships with parolees on their caseloads (Dowden and Andrews, 2004; Landenberger and Lipsey, 2005; Jolliffe and Farrington, 2007; Morash et al., 2014). Evidence-based practices in community corrections highlight the importance of practitioner-client relationships that are characterized by warmth, empathy, respect, and support (Dowden and Andrews, 2004; Ireland and Berg, 2007; Andrews, 2011). In these relationships, parole officers connect and collaborate with offenders, model pro-social behavior, communicate effectively, and apply motivational techniques (Walters et al., 2007). Positive parole officer-parolee relationships foster bonding, closeness, and trust between both parole officers and parolees (Ireland and Berg, 2007; Ross et al., 2008). These factors provide the necessary context in which change can happen. For example, interviews with parolees consistently demonstrate that positive relationships with their parole officers facilitate pro-social cognitive changes (e.g., identity shifts; Giordano et al., 2007; Bui and Morash, 2010; Morash et al., 2014; Stone et al., 2016) that can lead to stable, long-term success.

In contrast, negative parole officer-parolee relationships are characterized as authoritarian, unsupportive, inflexible, and controlling (Stone et al., 2016). Negative parole officer-parolee relationships have been found to be related to confrontation and noncompliance with the terms of supervision (Ireland and Berg, 2007; Morash et al., 2014; Chamberlain et al., 2017). Importantly, negative parole officer-parolee relationships are not conducive to pro-social cognitive changes (Morash et al., 2014; Stone et al., 2016). Taken together, this research suggests that the quality of the parole officer-parolee relationship plays a role in parolees’ cognitions and self-perception with implications for parolees’ overall success.

The extant research also begs the question, if experiences in the parole officer-parolee relationship has an impact on parolees’ cognitions and self-perception, can these experiences also impact the parole officers’ cognitions; especially their self-perception? Specifically, how do negative or positive parole officer-parolee experiences impact the way parole officers think about themselves in relation to those whom they supervise? The present study is the first to our knowledge to address this question by targeting implicit cognitions about the self in relation to parolees.

### Self-Expansion Theory and Implicit Social Cognition

Self-expansion theory posits that frequent and positive experiences with close others (e.g., spouses, close friends) can lead individuals to assume attributes, cognitions, and behaviors of those close others (Aron and Aron, 1986; Aron et al., 1991). Put differently, when the conditions of self-expansion are met, that is, frequent and positive experiences with a close other, an individual is more likely to associate their self-concept (i.e., identity and self-perception) with aspects of another person’s identity.

Self-expansion is based on individuals’ desire to enhance personal growth, progress, and self-efficacy (Aron and Aron, 1996). In close relationships, both individuals mutually include some or all aspects of the other into their self-concept. As a result, a mental overlap occurs between the self and the close other which allows both individuals to: (1) vicariously take on the resources (i.e., physical and social capital), (2) cultivate new perspectives, and (3) acquire new characteristics or identities related to the other and incorporate them into their self-concept (Aron and Aron, 1986, 1996, 1997; Aron et al., 1991, 1992). For instance, when a close other is perceived as a part of the self, the allocation of resources is shared (Clark and Mills, 1979), perspective differences are decreased (Brenner, 1973), and characteristics of others are perceived as one’s own (Tesser et al., 1988).

Self-expansion has been applied to understand the effects of intergroup relationships, such as cross-group friendships (e.g., Latino and White friendships; Page-Gould et al., 2010). This line of research suggests that people are motivated not only to expand their self-concept between close others as individuals but also the groups to which a close other belongs. Self-expansion in intergroup relationships requires that an individual has a relationship with a member of a different social group and that they engage in close, frequent, and positive experiences with each other, that then results in the motivation to associate with the group as a whole and its related traits (Smith and Henry, 1996; Coats et al., 2000; Aron and McLaughlin-Volpe, 2001).

Studies have demonstrated the self-expansion phenomenon using “explicit” or direct self-report measures (Aron et al., 1991, 1992; Page-Gould et al., 2010) and “implicit” or indirect (i.e., reaction time) measures (Aron et al., 1991; Aron and McLaughlin-Volpe, 2001; Page-Gould et al., 2010). For example, married couples exhibit explicit associations between the self and their spouse using the Inclusion of the Other in the Self (IOS) scale, a self-report measure of self-expansion on which participants indicate how close they perceive another person by selecting one of seven pairs of circles that vary in their between-circle distances to represent different degrees of cognitive overlap between the self and the other (Aron et al., 1992, 1995). Married individuals also demonstrate self-expansion on “implicit” measures (Aron et al., 1991; Aron and McLaughlin-Volpe, 2001). On “me/not-me” tasks in which reaction time is used to measure similarity, married participants are quicker (i.e., press a button labeled “me” versus one labeled “not me”) to categorize traits related to their spouses as self-descriptive than traits unrelated to their spouses. Similarly, on a “yes/no” task, participants in a romantic relationship respond faster (i.e., press a button labeled “yes” versus one labeled “no”) to traits relevant to both the participants and the partner than to traits that were different between themselves and their partner (Smith et al., 1999). Taken together, this past research provides evidence for explicit and implicit self-expansion.

### Professional Self-Expansion

Most of the self-expansion research has focused on self-expansion in personal relationships. To our knowledge, one study has examined self-expansion within the context of people’s occupations (McIntyre et al., 2014). McIntyre et al. (2014) employed a 14-item self-expansion questionnaire to measure the extent to which people exhibited self-expansion with their occupation as a whole. The study demonstrated that people can indeed self-expand with their occupation. Although McIntyre et al. (2014) focused on self-expansion with people’s occupations as a whole, they suggest that people can self-expand with others with whom they interact in the workplace if the interactions meet the pre-requisites of self-expansion. We extend their work and examine the extent to which parole officers self-expand with the group parolee, a group with which parole officers frequently interact in the workplace by measuring implicit criminal-self associations; one potential consequence of self-expansion with parolees.

### Implicit Self-Expansion in Parole Officers

The present study adopts an implicit social cognition approach utilizing an Implicit Association Test (IAT), to measure associations between the self-concept of parole officers with the social identity group criminal. Like other social identity groups, “criminals,” including parolees, self-categorize (Krueger, 2001) as criminal, and, as such, perceive themselves as sharing attributes, cognitions and experiences with the group (Boduszek et al., 2013). Further, past criminal experiences are sufficient for individuals to identify the self both explicitly (Asencio, 2011; Boduszek et al., 2013) and implicitly (Rivera and Veysey, 2015, 2018; Veysey and Rivera, 2017) with the group criminal. Indeed, repeated studies have established the IAT as a reliable and valid measure of implicit self-criminal associations (Rivera and Veysey, 2015, 2018; Veysey and Rivera, 2017). The present research, measuring the extent to which parole officers implicitly associate themselves with their parolees, extends past work on direct personal experience as a criminal to indirect personal experience with a member of the group criminal (i.e., parolee).

Our *a priori* hypothesis is that parole officers who have positive (versus negative) experiences with parolees are likely to exhibit relatively strong implicit associations with the group criminal. The logic underlying this hypothesis follows previous examinations of self-expansion. Parole officers may experience self-expansion through frequent and positive experiences with parolees. Indeed, the occupational role of parole officers requires them to frequently and directly meet with multiple parolees on a daily basis (at roughly 76 h per month; DeMichele, 2007). As such, these meetings provide opportunities for positive interactions that may foster a sense of closeness between parole officers and their parolees. Moreover, parole officers may view the success or shortcomings of their parolees as their own, reflecting a sense of interconnectedness. Finally, parole officers have the potential to engage in positive experiences with their parolees as they support parolees’ reintegration into society. For parole officers, therefore, it is not an experience within the criminal justice system that contributes to self-expansion and its effects on their mental associations with the group criminal, but rather their occupational experiences with others only who have had criminal justice experiences.

### Parole Officer’s Subjective Professional Identity and Orientation

The implicit cognitive consequences of the parole officer-parolee relationship to some degree may be dependent on parole officers’ professional characteristics, particularly the importance of, and their basic orientation toward, their role (Seiter and West, 2003; Walters et al., 2007). Consistent with role identity theory, roles that individuals take on, such as occupation, theoretically should affect officers’ self-concept and cognitions (Stryker and Burke, 2000), especially on the job where the role is most likely to be highly salient. Two aspects of parole officers’ role are professional orientation and subjective professional identification.

Parole officer orientation has been measured along a continuum ranging from surveillance and strict law enforcement to therapeutic support (Sigler and Mcgraw, 1984; Seiter and West, 2003). Research suggests that professional orientation affects the quality of experiences within the parole officer-parolee relationship (Skeem et al., 2003, 2007; Kennealy et al., 2012; Blasko et al., 2015). Surveillance restricts and controls parolee behavior to ensure that individuals fulfill the responsibilities and conditions of parole (Fulton et al., 1997; Seiter, 2002; Skeem et al., 2003). Also, surveillance is related to low levels of trust and cooperation, which may be related to negative experiences within the parole officer-parolee relationship (Fulton et al., 1997; Seiter, 2002; Skeem et al., 2003). Conversely, parole officers who take on a therapeutic role may be more likely to have positive experiences with their parolees as this orientation requires the parole officer to deeply engage with parolees, and to aid them in addressing criminogenic obstacles, such as mental health problems, substance abuse, physical health conditions, inadequate educational and employment skills, and lack of stable housing (Seiter, 2002; Petersilia, 2003; Travis, 2005; National Research Council, 2008; Tatman and Love, 2010; Blasko et al., 2015). Therefore, professional orientation may moderate the effect of parole officers’ relationship experiences with parolees on their implicit associations with parolees. Specifically, among parole officers who take on a therapeutic orientation, a positive experience with a parolee may yield stronger implicit associations with parolees in comparison to those who do not take on a therapeutic orientation.

In addition, parole officers vary in the extent to which they identify with their occupational group. According to social identity theory, people often identify with the social groups to which they belong, including occupational groups (Hogg and Turner, 1987). The extent to which people identify with their occupational group may reflect commitment (Ellemers and Rink, 2005) and is related to positive workplace behavior such as job performance (Meyer et al., 2002; Becker and Kernan, 2003). By extension, the extent to which people identify as a parole officer may influence the types of experiences parole officers have with parolees. For example, those who strongly identify as a parole officer may be more committed to assisting parolees in successful reentry, in turn, impacting their experiences with parolees. Therefore, the extent to which they identify with their occupational group may moderate the effect of parole officers’ relationship experiences with parolees on their implicit associations with parolees. Specifically, among parole officers who strongly identify with their occupational group, a positive experience with a parolee may yield stronger implicit associations with parolees in comparison to those who do not strongly identify with their occupational group.

### The Present Study

This experiment is the first of its kind to examine implicit associations between the self-perception of criminal justice practitioners, specifically parole officers, and individuals in the criminal justice system. We experimentally manipulated parole officers’ memory of either a positive or negative experience with a parolee, then utilized an IAT to measure implicit self-criminal associations. We tested the *a priori* main hypothesis that officers who are reminded of a positive experience with a parolee will exhibit stronger implicit associations with the group criminal in comparison to parole officers who are reminded of a negative experience. Finally, we explored the moderating effect of parole officer orientation and parole officer group identification on the relation between the manipulation (i.e., positive or negative memory task) and implicit self-criminal association.

## Materials and Methods

### Participants and Design

The population of interest were the 258 parole officers in the New Jersey State Parole Board Division of Parole. At the time of the experiment, the parole officers averaged caseloads of approximately 50 parolees per officer. Officers were expected to conduct three face-to-face interactions per month, including one home visit, per individual. However, it is important to note that these were the minimum standards per parolee, and officers had the discretion to meet with parolees as frequently as deemed fit given the circumstances. Officers had the ability to interact with parolees in a variety of contexts ranging from the parole office to counseling locations to transporting parolees to important appointments.

We invited all New Jersey State parole officers to participate; first *via* an on-line platform and then through face-to-face invitations to officers on duty at district offices. Eighty-seven (*n* = 18 online; *n* = 69 district office) active parole officers completed the experiment. All data were collected anonymously, and participants volunteered to complete the study without any incentive. Three participants’ data were excluded from analyses due to reaction time error rates on the IAT that were greater than 30% overall or over 40% on any given block as recommended by Greenwald et al. (2003).

The final sample consisted of 84 parole officers (28 females, 55 males, 1 other,^1^ *M*_age_ = 37.10, SD_age_ = 7.39, age range: 25–54 years). Table 1 lists the demographics and characteristics of the final sample. Approximately 49% percent of officers identified as White, 26% were Hispanic, 13% were Black, 11% were another ethnicity not listed, and 1% identified as Asian or Pacific Islander. On average, parole officers had been working in their position for nearly nine years (*M*_yearsparole_ = 8.64, SD_yearsparole_ = 6.634, range: 1–25 years). Approximately 51% of officers were from the sex offender management unit, 35% of parole officers were from a traditional unit, and 14% were from other units. Approximately 18% of parole officers had a criminal history (i.e., arrest, conviction, and/or incarceration). The experiment employed a one-factor two-level (parole officer-parolee experience condition: positive versus negative) between-participants design.

**Table 1:** Demographics and characteristics of sample participants (*N* = 84).

Variable	%	*M* (SD)
Gender		
Male	65.5	–
Female	33.3	–
Age	–	37.10 (7.39)
Race/ethnicity		
White	48.8	–
Hispanic/Latino	26.2	–
African American	13.1	
Other	10.7	–
Asian or Pacific Islander	1.2	–
Years as PO	–	8.6 (6.63)
Caseload type		
Sex Offender Management Unit	51.2	–
Traditional	34.5	–
Other	14.3	–
Criminal history	17.9	–

### Manipulated Variable

#### Positive Versus Negative Memory Writing Task

Rooted in the basic principles of self-expansion theory, the purpose of the manipulation was to make salient an experience parole officers have had with a parolee. Parole officers were randomly assigned to vividly re-experience either a positive or a negative experience with a parolee. Participants in the positive experience condition were given the following prompt: “Please imagine a positive interaction you have had with a parolee. Please describe the positive experience as well as your thoughts and feelings during this positive interaction. Please provide details and write freely.” Participants in the negative experience condition were given the same prompt, but were asked to focus on a negative interaction. Fifty-two percent of parole officers were randomly assigned to the positive experience condition and 48% to the negative experience condition.

A review of the writing responses confirmed that participants responded to the prompt with positive responses (i.e., used positive words) in the positive experience condition and negative responses (i.e., used negative words) in the negative experience condition, with two exceptions. Two participants (2%) provided a mixed response, one in each condition. Also, we conducted an ANOVA to test if the length of responses (i.e., the number of words) between conditions were different. There was no difference, on average, between the positive (*M =* 66.65, SD *=* 64.11) and negative (*M =* 65.50, SD = 50.44) conditions, *F*(1, 82) = 0.008, *p* = 0.928.

### Measured Variables

#### Implicit Criminal-Self Associations

The current study used a Single-Category Implicit Association Test (SC-IAT; Greenwald et al., 1998; Karpinski and Steinman, 2006), which uses reaction times to operationalize the strength of implicit mental associations between the self and criminal (Rivera and Veysey, 2015, 2018; Veysey and Rivera, 2017). Parolees are members of the group criminal. Prior to becoming a parolee, a person must first be convicted of a crime and incarcerated, and, therefore, is formally labeled a criminal. Also, during a first introduction, parole officers are made aware of a parolee’s criminal history, thus affirming the parolee’s identity as a criminal.

The SC-IAT was administered on a computer and participants were asked to complete four blocks of reaction time trials that were preceded by a set of instructions. Semantic stimuli that represent self, other, and criminal randomly appeared one after the other in the center of the screen. The self-related words were *me, my, mine, I,* and *myself*. The other-related words were *they, them, their, theirs,* and *other* (“self” and “other” words were used in prior studies, e.g., Rivera and Veysey, 2015; Veysey and Rivera, 2017). The criminal-related words were *criminal, felon, lawbreaker, offender, convict, delinquent,* and *prisoner*. The criminal words were pre-tested with a separate adult sample (for a full description, see Rivera and Veysey, 2018).

As each word appeared on the screen, category labels were positioned on the top left and top right of the screen. For half of the task, participants used the “A” key to classify the words that belong to either the “self” or “criminal” category (labels on the top left) and the “K” key to classify the words that belong to the “other” category (label on the top right). The second half of the task was reversed; participants used the “A” key to classify the words that belong to the “self” category (label on the top left) and the “K” key to classify the words that belong to the “other” or “criminal” category (labels on the top right). These tasks were counterbalanced between participants. For each task, participants first read the instructions then completed 17 practice trials, followed by 51 critical trials. For each trial, the target word remained on the screen until participants classified it to one of the three categories on the monitor (“self,” “other,” or “criminal”). If the participant responded correctly, a new target word appeared. If the participant responded incorrectly, the message “ERROR” appeared on the screen in place of the target word and remained until the participant pressed the correct key.

The SC-IAT was scored in accordance with the procedures outlined by Greenwald et al. (2003) and Karpinski and Steinman (2006). The score is the difference between the reaction times between the self and criminal trials and the other and criminal trials. Relatively higher SC-IAT scores indicate faster reaction times when self-related words and criminal-related words are paired together than when other-related words are paired with criminal words. Thus, a higher IAT score indicates stronger associations between self and criminal.

#### Subjective Identification With Parole Officers

Participants indicated the extent to which they identified with their professional group. Parole officers were asked to think about their identification with other parole officers and respond to two items (“Being a parole officer is an important part of who I am” and “Being a parole officer is important to my sense of self”) on a 7-point scale ranging from *strongly disagree (0)* to *strongly agree (6).* The two items were highly correlated (*r* = 0.75, *p* < 0.001) and therefore combined into a single measure.

#### Professional Orientation

Guided by the Parole Officer Professional Orientation measure (Fulton et al., 1997), which was designed to assess how parole officers perform their job functions and goals, participants indicated the extent to which they adopted a professional orientation (i.e., a surveillance vs. therapeutic orientation). Parole officers were asked to rate how they perform their job and respond to two items: (a) one regarding their subjective job role—“The most important aspect of your job is …” on a 5-point scale ranging from *exclusively social work (1)* to *exclusively law enforcement (5)*; and (b) a second regarding their occupational strategy: “The most effective way to change behavior is through…” on a 5-point scale ranging from *exclusively positive reinforcement (1)* to *exclusively punitive sanctions (5).* Higher scores on both questions indicate a stronger focus on strategies related to law enforcement. While both questions sought to measure the construct of professional orientation, their low correlation, *r*(84) = 0.39, *p* < 0.001, suggests that they are tapping into different elements of professional orientation. Therefore, the items were not combined and were explored independently.

#### Demographics

Participants completed a demographics and background questionnaire that included variables such as gender, age, race/ethnicity, length of time as a parole officer, type of caseload, and personal criminal history.

### Procedure

Participants were informed that the study’s purpose was to examine the relation between parole officers’ professional experiences and attitudes. First, participants were randomly assigned to and completed the positive or negative experience writing task and then completed all remaining measures in the order listed above. Finally, participants were fully debriefed about the purpose of the study.

## Results

See Table 2 (entire sample) and Table 3 (by experience condition) for descriptives and zero-order correlations.^2^

**Table 2:** Zero-order correlations and descriptives for all participants (*N* = 84).

	1	2	3	4	5	6
1. Implicit criminal-self association	–					
2. Subjective parole officer identification	−0.13	–				
3. Subjective job role	−0.04	0.12	–			
4. Occupational strategy	−0.18	−0.12	0.39^*^	–		
5. Age	−0.02	−0.39^*^	−0.01	−0.14	–	
6. Years as PO	−0.06	−0.48^*^	0.06	0.05	0.67^*^	–
*M*	−0.09	4.40	3.15	2.87	37.10	8.64
SD	0.15	1.41	0.63	0.58	7.39	6.63

* *p* < 0.01.

**Table 3:** Zero-order correlations and descriptives by experience condition.

	1	2	3	4	5	6	*M*	SD
1. Implicit criminal-self association	–	−0.03	−0.09	−0.02	−0.22	−0.24	−0.05	0.14
2. Subjective parole officer identification	−0.22	–	−0.09	−0.19	−0.43^**^	−0.57^**^	4.40	1.34
3. Subjective job role	−0.06	0.31^*^	–	0.46^**^	0.04	0.07	3.23	0.64
4. Occupational strategy	−0.29	−0.04	0.35^*^	–	0.85	0.87	2.82	0.54
5. Age	0.13	−0.36^*^	−0.11	−0.23	–	0.71^*^	37.95	7.74
6. Years as PO	0.14	−0.39^*^	0.07	0.07	0.66^**^	–	8.25	6.55
*M*	−0.13	4.41	3.08	2.93	36.15	9.08	–	–
SD	0.15	1.51	0.62	0.62	6.95	6.79	–	–

Numbers above the diagonal are data from participants in positive experience condition (*n* = 44). Numbers below the diagonal are data from participants in negative experience condition (*n* = 40).

* *p* < 0.05;

** *p* < 0.01.

To test our main hypothesis of the effect of making parole officer experiences salient on implicit associations, SC-IAT scores (criminal-self association strength) were subjected to a one-way analysis of variance (ANOVA). As predicted, participants who were reminded of a positive experience with their parolees (*M* = −0.05, SD = 0.14) exhibited stronger associations between criminal and self when compared to those who were reminded of a negative experience with their parolees (*M =* −0.13, SD = 0.15), *F*(1, 82) = 5.20, *p* = 0.025, *d* = 0.50 (medium effect size).^3^ These findings support our hypothesis that parole officers who are reminded of positive experiences exhibit a stronger implicit association (i.e., a reduced cognitive distance) between criminal and self in comparison to parole officers who are reminded of negative experiences.

We next explored if individual differences in subjective group identification or the two professional orientations (i.e., subjective job role and occupational strategy) moderated the effect of past experiences on criminal-self associations. To this end, we conducted three hierarchical multiple regression analyses in which scores on the three individual differences measures (mean-centered) and parole officer-parolee experience condition (coded 0 = negative experience, 1 = positive experience) were entered in the first step and their interaction term in the second step. Consistent with our main hypothesis results above, the main effect of the Experience Condition was significant across the three models, Δ*F*s(2, 81) > 2.81, *p*s < 0.036, *R*^2^s > 0.07, *β*s > 0.23, *p*s < 0.025. However, there was no significant main effect of Subjective Parole Officer Identification, Δ*F*(2, 81) = 3.29, *p* = 0.247, *R*^2^ = 0.08, *β* = −0.13, *p* = 0.247, and no significant Subjective Parole Officer Identification X Experience Condition interaction, Δ*F*(3, 80) = 0.66, *p* = 0.417, *R*^2^ = 0.08, *β* = 0.12, *p* = 0.417; no significant main effect of Subjective Job Role, Δ*F*(2, 81) = 2.82, *p* = 0.493, *R*^2^ = 0.07, *β* = −0.08, *p* = 0.693, and no significant Subjective Job Role X Experience Condition interaction, Δ*F*(3, 80) = 0.01, *p* = 0.929, *R*^2^ = 0.07, *β* = −0.01, *p* = 0.929; and, finally, no significant main effect of Occupational Strategy, Δ*F*(2, 81) = 3.72, *p* = 0.146, *R*^2^ = 0.08, *β* = −0.16, *p* = 0.063, and no significant Occupational Strategy X Experience Condition interaction, Δ*F*(3, 80) = 1.40, *p* = 0.241, *R*^2^ = 0.10, *β* = 0.17, *p* = 0.241.

## General Discussion

This research extends earlier findings regarding implicit self-expansion in general, contributes to the sparse literature on employment-based self-expansion, and provides evidence of a self-expansion mechanism to a highly stigmatized group. The study examined the extent to which parole officers implicitly associate self with the group criminal as a function of a memory-based manipulation. The primary finding that officers who are reminded of a positive experience with a parolee demonstrate stronger implicit self-criminal association on the IAT than those who were reminded of a negative experience with parolees supports other group-based implicit self-expansion studies. These former studies, however, target one or more cross-group friends and self-expansion to their groups (e.g., having one or more Latinx friends and the group Latinxs; Page-Gould et al., 2010). The present study, like McIntyre et al.’ (2014), is based within a professional work environment. While McIntyre et al. (2014) used “job” as a proxy group identity, the present research tested self-expansion to the client group of parolees after manipulating a memory in which officers imagined an interaction with an individual parolee on their caseload.

For self-expansion at the group level to occur, interactions with one or more members of a group must be: (1) close, (2) frequent, and (3) positive. We suggest that these self-expansion requirements occur in parole officers’ relationships with a parolee in the workplace. Closeness is promoted through their relationship interactions with individual parolees who represent the group and its characteristics. The role of the parole officer requires that they interact with parolees for numerous hours a week. During this time parole officers engage with parolees and support them to lead pro-social lives (e.g., assistance with treatment, job placement, and housing). High frequency is assumed due to the time spent and average number of parole officer-parolee interactions on any given day. And, finally, positive (vs. negative) interactions were experimentally manipulated in the present research. Under these conditions, the results demonstrated that a single recalled positive versus negative memory had a significant effect on implicit associations between parole officers’ self and the group criminals.

While our main hypothesis was supported, the nature of the effect needs further understanding. Self-expansion is about taking on aspects of the close other or of the close other’s group. In our research, we infer evidence for self-expansion from parole officers’ stronger implicit self-criminal associations on the IAT following the reminder of a past positive relative to a negative experience with a parolee. The mean IAT score in the positive experience condition, however, was near the midpoint of the scale, whereas the mean IAT score in the negative experience condition significantly more. Since the IAT in general is a measure of relative associations, presently self-criminal associations relative to other-criminal associations, its scoring is a function of the difference between reaction times to categories simultaneously paired on the computer screen. Thus, another yet complementary suggestion from our data is that parole officers in the negative experience condition increased their cognitive distance between their self-concept and the group parolee, whereas those in positive experience condition demonstrated a decrease in this cognitive distance.

The potential positive impact of these findings is related to the malleability of this effect. If a single positive reminder affects implicit associations between the officer and the parolee, the quality of the parole officer-parolee relationship may be similarly affected. In general, self-expansion results in the acquisition of perspectives, causing the self to be concerned with the needs of the other (Wegner, 1980; Deutsch and Mackesy, 1985; Aron and Aron, 1986). Thus, even in relationships with people belonging to stigmatized groups, self-expansion results in the reduction of cognitive distance. Within this definition, self-expansion may be a cognitive driver of empathy, which requires one to take the perspective of the other (Selman, 1980; Wegner, 1980; Aron et al., 2004; Batson, 2009).

Empathy is an essential component of the therapeutic relationship, and it can facilitate collaboration, trust, and understanding between the practitioner and client (Murphy and Baxter, 1997) and is related to positive client outcomes (Taxman, 2002; Ross et al., 2008; Taxman and Ainsworth, 2009; McCambridge et al., 2011). Empathy is also related to bonding, feelings of closeness, attitudes towards stigmatized groups (Finlay and Stephan, 2000), and changes in behavior, such as helping the other (Batson et al., 2002). Therefore, it is plausible that empathy follows from self-expansion and, as such, positive reminders and messaging may be used to improve the quality of the interaction and, thus, improve parolee success (Gunnison and Helfgott, 2011).

Although the present study does not directly measure empathy, our data suggest that one way to potentially bolster empathy between parole officers and parolees is to provide environments that foster positive interactions. Practices such as Motivational Interviewing have been increasingly adopted by corrections agencies due to their positive effects on parolees (Dowden and Andrews, 2004; Landenberger and Lipsey, 2005), and have an effect on parole officers as well (Iarussi and Powers, 2018). Because Motivational Interviewing promotes rapport and requires the parole officer to take the perspective of the parolee, it is therefore possible that Motivational Interviewing also provides parole officers with opportunities to acquire the perspective of their parolees and promote helping behaviors that support parolees’ successful reentry and long-term desistance.

In the absence of these experiences, reminding parole officers of positive or successful experiences with parolees may serve to strengthen implicit self-criminal associations, which can have occupational behavioral effects. Positive experiences can be promoted through the use of bulletin boards that showcase events in which parole officers and parolees work together or celebrate the accomplishments of parolees (e.g., educational or employment successes). This practice may serve as a reminder that successful and positive experiences between parole officers and parolees do happen and can result in positive outcomes. Future research should examine the relation between implicit self-criminal associations and empathy and its downstream consequences for parole officer behaviors and other outcomes.

Additionally, we explored the moderating role of parole officers’ subjective professional identity (i.e., professional orientation and parole officer identification) on the relation between past experiences and implicit self-criminal associations. None of the measures of subjective professional identity were related to implicit associations, nor did they moderate the effect of past experiences on implicit associations. This suggests that making a positive memory salient is powerful enough to override (i.e., regardless of) individual-level professional characteristics.

While self-expansion may impact parole officer performance and parolee outcomes, it is important to note that the present study cannot answer the fundamental question about whether implicit cognitions in this setting affect parole officer behavior. To be clear, this study is not longitudinal nor does it include measures of actual parole officer behavior or parolee success. However, research suggests that self-expansion can influence not only cognition but also behavior (Aron et al., 1991; Aron and Aron, 1996; Davis et al., 1996; Cialdini et al., 1997). Moreover, implicit social cognitions predict behavioral actions, often having greater explanatory power in behavioral outcomes than explicit social cognitions (Greenwald et al., 2009; Charlesworth and Banaji, in press). Further, research demonstrates that occupations which promote self-expansion are related to increased job satisfaction and commitment (McIntyre et al., 2014). Based on prior work, we speculate that self-expansion may have implications for the well-being of parole officers as well.

This study utilizes data from parole officers, a unique and often inaccessible sample. However, obtaining data from this sample is not without its limitations. New Jersey employs nearly 600 parole officers across 16 parole offices throughout the state (New Jersey State Parole Board, 2020). First, the sample size was relatively small due to the time consuming and costly nature of in-person data collection from on-duty officers during department-wide meetings. The researcher responsible for data collection visited several parole offices numerous times over the course of two years. The intention was to achieve a high response rate. However, some officers were not willing to participate in this research for various reasons ranging from lack of interest to job demands. Ideally, future research with a larger sample could expand on the current research. Second, because the study was limited to ten minutes per participant, the amount of data that could be obtained was limited. For example, measures of explicit association were not collected. While implicit social cognition research demonstrates that implicit and explicit associations with stigmatized groups are often poorly correlated or uncorrelated (Greenwald et al., 2009), self-expansion research demonstrates that implicit and explicit associations are often correlated (Coats et al., 2000; Page-Gould et al., 2010). Future research should examine the effect of experiences on both implicit *and* explicit associations to provide a comprehensive understanding of self-expansion with this particular group.

Finally, while this study examines the effect of reminders of past experiences with a single parolee, this study does not answer the question of repeat experiences. Do parole officers who have a positive experience with a parolee tend to repeatedly engage in positive experiences with their parolees? If so, parole officers who frequently engage in positive experiences may exhibit self-expansion even in the absence of contexts which facilitate positive experiences, and, therefore, translate to continuous positive experiences with parolees. This is squarely in line with research that demonstrates that individuals who had high quality relationships with cross-group friends did not exhibit stress or anxiety following conflict (Page-Gould, 2012). For parole officers, negative experiences with parolees may occur, but parole officers who self-expand may be better equipped to overcome such experiences, thereby potentially improving their relationships with parolees. Despite these limitations, the present data suggest a promising step in understanding the conditions which strengthen implicit associations with parolees.

## Conclusion

The present study is the first experiment at the intersection of psychology and criminology to apply the self-expansion model and implicit social cognition measurements within a criminal justice setting to understand the cognitive effects of a parole officer’s relationship with parolees. Results demonstrate that parole officers who are reminded of positive experiences with a parolee exhibit stronger associations between themselves and the group parolee in comparison to those reminded of a negative experience. Positive experiences can increase parole officers’ positive perceptions of parolees, as well as bolster their overall working relationship with parolees. This may lead to benefits such as increased job satisfaction and may have downstream consequences for parolees; namely, desistance and successful reentry – a primary goal of parole and of the criminal justice system.

## Data Availability Statement

The raw data supporting the conclusions of this article will be made available by the authors, without undue reservation.

## Ethics Statement

The studies involving human participants were reviewed and approved by Rutgers University IRB. The patients/participants provided their written informed consent to participate in this study.

## Author Contributions

All authors listed have made a substantial, direct, and intellectual contribution to the work and approved it for publication.

## Funding

This research was partially supported by the Rutgers School of Criminal Justice and the Rutgers Implicit Social Cognition (RISC) Lab.

## Conflict of Interest

The authors declare that the research was conducted in the absence of any commercial or financial relationships that could be construed as a potential conflict of interest.

## Publisher’s Note

All claims expressed in this article are solely those of the authors and do not necessarily represent those of their affiliated organizations, or those of the publisher, the editors and the reviewers. Any product that may be evaluated in this article, or claim that may be made by its manufacturer, is not guaranteed or endorsed by the publisher.

## Footnotes

^1^One participant did not indicate their gender.

^2^Zero-order correlations between the demographic variables (age, length of time as a parole officer), subjective parole officer identification, the professional orientation measures (subjective role, occupational strategy), and implicit criminal-self association scores were conducted for the entire sample and by each condition. The relation between the categorical demographic variables (gender, race/ethnicity, type of caseload, criminal history, district office location) and implicit criminal-self association scores across the entire sample and by condition were analyzed using a series of ANOVAs. For gender, participants who identified as “other” were nominal (*n* = 1), so gender was coded was coded (1 = *male;* 0 *= female, other*) to facilitate the interpretation of the results; race/ethnicity was coded (1 = *African American/Black and Hispanic*; 0 = *all others*), type of caseload was coded (1 = *sex offender unit*; 0 = *all other units*), criminal history was coded (1 = *arrested, convicted, and/or incarcerated*; 0 = *was not arrested, convicted, and/or incarcerated*), and district office location was coded (1 = *online participant/unknown district*; 2 = *Northern New Jersey*, 3 = *Central/Southern New Jersey*). The ANOVAs and zero-order correlation analyses revealed that no demographic and measured variables were statistically significantly related to implicit criminal-self association scores.

^3^We conducted the same analyses using the subsample of participants who did not have a criminal history (*n* = 69). Results were similar to those with the full sample (positive experience: *M = −*0.05, SD = 0.15, negative experience: *M* = −0.12, SD = 0.16; *F*(1, 67) = 4.11, *p* = 0.047, *d* = 0.45, medium effect size).
